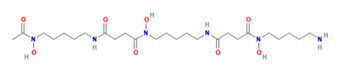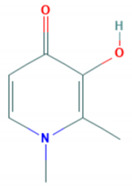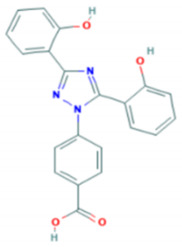# Iron Chelation in Local Infection

**DOI:** 10.3390/molecules26010189

**Published:** 2021-01-02

**Authors:** Cassidy Scott, Gaurav Arora, Kayle Dickson, Christian Lehmann

**Affiliations:** 1 Department of Anesthesia Pain Management and Perioperative Medicine, Dalhousie University, Halifax, NS B3H4H7, Canada; gr395187@dal.ca (G.A.); kayle.dickson@dal.ca (K.D.); chlehmann@dal.ca (C.L.); 2 Department of Pharmacology, Dalhousie University, Halifax, NS B3H4H7, Canada; 3 Department of Medicine, Dalhousie University, Halifax, NS B3H4R2, Canada; 4 Department of Microbiology and Immunology, Dalhousie University, Halifax, NS B3H4H7, Canada

**Keywords:** iron, chelation, local infection, siderophores

## Abstract

Iron is an essential element in multiple biochemical pathways in humans and pathogens. As part of the innate immune response in local infection, iron availability is restricted locally in order to reduce overproduction of reactive oxygen species by the host and to attenuate bacterial growth. This physiological regulation represents the rationale for the therapeutic use of iron chelators to support induced iron deprivation and to treat infections. In this review paper we discuss the importance of iron regulation through examples of local infection and the potential of iron chelation in treating infection.

## 1. Introduction

**Figure 1:**
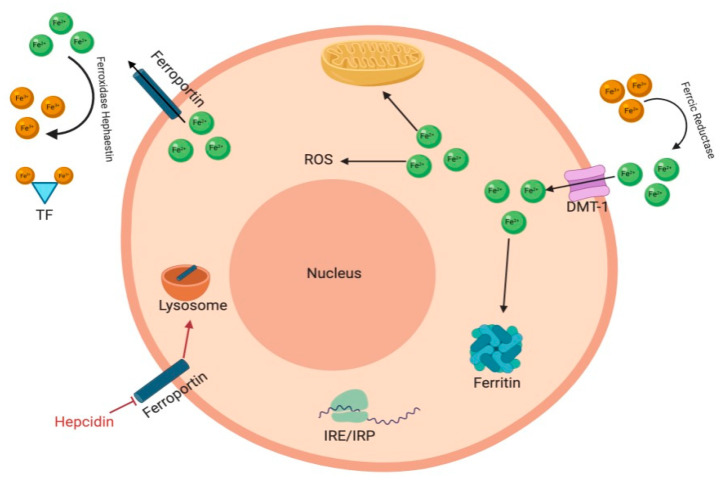
Iron Regulation. Iron is reduced by ferrireductase and then transported through by the divalent metal transporter 1 (DM1). Intracellularly, free iron catalyzes the production of reactive oxygen species (ROS). Ferritin stores any excess iron in the intracellular space, oxidizes ferrous iron and sequesters this ferric iron into a ferrihydrite mineral core. Intracellular iron levels are regulated through the iron-responsive element/iron-regulatory protein (IRE/IRP). IRP 1 and IRP 2 bind to IRE on mRNAs that regulate iron metabolism. Ferroportin exports ferrous iron into the plasma after which it is oxidized by ferroxidase hephaestin and is bound to transferrin (TF). Hepcidin is a peptide hormone and a regulator of iron. It causes ferroportin to be internalized and degraded in lysosomes. Figure generated with bioRender.

Iron is an essential element for almost all living organisms as it plays a role in DNA synthesis, transcription, and repair as well as other important functions such as oxygen transport, cellular respiration, and immune response [1]. In humans, about 65% of the total iron is allocated in red blood cells (RBCs), 14% in macrophages, 10% as myoglobin in muscle fibers, 6% is stored in the liver, and 5% in bone marrow. Most of the iron is found within heme in RBC hemoglobin, used to distribute oxygen to every cell in the body. Mature RBCs have a life span of approximately 120 days, after which they are phagocytosed by specialized macrophages and the iron recycled [2]. Heme iron is mainly found within meat in the form of hemoglobin and myoglobin. In the stomach, heme is released from these proteins due to the low gastric pH and the action of proteolytic enzymes. Concentrated heme produced from hemoglobin hydrolysis is poorly soluble at a low pH. However, heme solubility is increased by the presence of protein. Therefore, the peptides and amino acids produced from meat hydrolysis can aid in enhancing the absorption of heme [3]. Inorganic, nonheme iron is available in many foods and is absorbed at the intestinal brush border by duodenal enterocytes. Using different pathways, heme and non-heme iron pass from the intestinal lumen to enterocytes across the brush border. Heme iron is taken up into the enterocyte as the intact metalloporphyrin by the heme carrier protein 1 (HCP1) [4]. Nonheme iron is reduced to Fe^2+^ by ferrireductase, cybrd1 (DcytB) prior to transportation through the cellular membrane by the divalent metal transporter 1 (DMT1) [5]. Excess intracellular iron is stored in the storage protein, ferritin. In the plasma, iron circulates bound to the glycoprotein transferrin (Tf), which has two binding sites for ferric iron [1]. Iron levels are tightly regulated through the process of utilization, recycling, and storage to avoid iron overload or deficiency. Hepcidin, a peptide hormone secreted by the liver, together with its main target, the iron exporter protein ferroportin, are the most important physiological regulators of systemic iron levels [6]. Hepcidin causes ferroportin to be internalized and degraded in lysosomes. However, hepcidin can also be released to regulate iron levels locally by cells such as macrophages and neutrophils. Intracellular iron levels are regulated through the iron-responsive element/iron-regulatory protein (IRE/IRP). Here IRP 1 and IRP 2 bind to IRE on mRNAs that regulate iron metabolism [1] (Figure 1). Proteins of iron metabolism are regulated post-transcriptionally by intracellular iron levels through the action of IRP. Cytoplasmic IRP binds to specific mRNA stem loop structures known as IRE. Under conditions of iron depletion IRP bind to IRE at the 3′-untranslated regions (UTR) of transferrin receptor 1 (TFR1) and DMT1 mRNA to increase both transcription and protein levels. In contrast, IRP can also bind to the 5′UTR of ferritin mRNA inhibiting translation of ferritin mRNA, causing ferritin protein levels to decrease. When there is excess iron, RNA-binding activity of IRPs is lost, and they fail to bind IRE. Therefore, translation of ferritin mRNA is stimulated, and ferritin protein levels rise [4].

Iron is able to exist in two oxidation states: Fe^2+^ (ferrous) and Fe^3+^ (ferric) [1]. Through the Fenton and Haber–Weiss reactions, free iron catalyzes the production of reactive oxygen species (ROS) [7]. ROS generation is beneficial during infection as it aids in destroying microbes. However, over production of ROS due to the presence of excess iron can result in oxidative stress and damage DNA, lipids, and proteins [8]. Therefore, in local infection excess iron is detrimental to the host.

The regulation of iron metabolism is important during any type of infection since iron is also an essential requirement for microorganisms. In response to local infections monocytes circulate to the tissue and release hepcidin through a toll-like receptor 4 (TLR-4) dependent pathway [9]. The release of autocrine hepcidin from monocytes results in decreased expression as well as internalization and degradation of ferroportin. As a result increased iron retention is observed within macrophages [10]. Stimulation of other pathways such as TLR2/TLR6 and interferon-gamma have been shown to be involved in reducing ferroportin expression through hepcidin-independent and dependent mechanisms, respectively [11,12]. Furthermore, lactoferrin, a host glycoprotein that binds iron with high affinity is found in mucosal secretions and released by specific granules of neutrophils in response to cytokines at sites of local infection [13].

In this paper we review the potentially detrimental effects of iron through examples of local infection such as microbial keratitis, skin wound infection and bacterial cystitis and examine the potential role of iron chelation as a novel treatment.

## 2. Iron Homeostasis

Iron is an essential trace element. However, the ability of iron to cycle between the oxidized and reduced forms can result in the formation of reactive oxygen species. Therefore, organisms have developed elaborate mechanisms to carefully regulate iron metabolism. Iron enters the body through the diet as either heme or nonheme iron. Heme iron is well absorbed, found mainly in meat, poultry, and fish. Non-heme iron is not absorbed as easily and comes mainly from plants. Over 95% of functional iron in the human body is in the form of heme [3]. Nonheme iron can be bound by dietary components which impedes its absorption, such as phytates and polyphenols. However, heme iron is tightly sequestered within a protoporphyrin ring, allowing for more efficient absorption as its absorption cannot be hindered by dietary components [14].

Iron is absorbed by mature enterocytes in the small intestine. Once iron has been taken up by enterocytes it can then be transported into the bloodstream for use in other tissues or stored inside endogenous ferritin. In order to be transported from the intestine into the blood stream, iron must cross the apical brush-border membrane and the basolateral membrane of enterocytes. Once within the enterocyte, iron is released from heme through heme oxygenase. On the brush border, ferric reductase activity facilitates the reduction of dietary ferric nonheme iron to the ferrous forum which can be transported via the ferrous iron transporter DMT-1. If the iron is not immediately needed it is stored in the cell within the storage protein ferritin. Once required iron can be transported across the enterocyte basolateral membrane via ferroportin (FPN1). Ferroportin is coupled with hephaestin, a ferroxidase which functions to convert ferrous iron to ferric iron [15].

Iron is supplied to the bloodstream through erythrocyte degradation, duodenal enterocytes absorbing dietary iron and hepatic stores. These processes all release iron into the circulation via the only known iron exporter, ferroportin. Ferroportin is regulated by hepcidin, a peptide that binds ferroportin and induces its internalization and degradation by proteasomes. Iron in excess to the bodies requirements is stored mainly in hepatocytes. The liver monitors iron levels and secretes hepcidin when levels are adequate or too high in order to decrease iron release into the bloodstream [16]. Hepcidin is produced at a high rate (10 mg/d) at baseline and is cleared rapidly from the circulation by the kidneys, with a half-life of several minutes. This allows for hepcidin levels to change rapidly in response to changing blood plasma iron concentrations, liver iron stores, inflammation, and erythropoiesis [17].

Once in the circulation, iron is bound to plasma transferrin. Each transferrin molecule can bind up to two atoms of iron. Diferric transferrin delivers iron to cells by binding to transferrin receptor (TfR) 1 on the plasma membrane. The transferrin- TfR1 complex is internalized via endocytosis and a reduction of transferrin-bound Fe^3+^ allows for the release of iron from transferrin. Iron can then move into the cytoplasm across the endosomal membrane via DMT1 and be used by the cell [14].

The majority of iron in the body is found within heme in hemoglobin. Mature erythrocytes have a life span of 120 days, after which they will be phagocytosed by macrophages that recycle iron. Recycled iron from erythrocyte degradation accounts for over 90% of daily iron requirement in humans. FPN1 is highly expressed in macrophages and pumps ferric iron into the circulation for recycling [18].

Ferritinophagy is an iron-dependent physiological process in which ferritin is degraded and iron is released. This process is mediated by nuclear receptor coactivator 4 (NCOA4). When NCOA4 levels are deplete, iron availability is reduced and iron-responsive element-binding protein 2 (IRP2) activity is induced to promote the translation of transferrin receptor. [17]. NCOA4-mediated ferritinophagy maintains intracellular iron homeostasis by facilitating ferritin iron storage or release, depending on iron availability and demand. Decreased NCOA4 levels inhibit ferritinophagy and increase ferritin iron storage [19].

Ferritinophagy also plays a role in the modulation of ferroptosis. The ability of iron to easily switch between Fe^2+^ and Fe^3+^ forms renders iron as the major catalyst for generation of reactive free radicals. For this reason, iron is tightly bound to transferrin, keeping it in the redox-inert state. Non-transferrin bound iron can accumulate in the plasma during pathological states and play a role in ROS generation. Excess ROS are detoxified by antioxidants. However, imbalance in the rate of ROS generation and detoxification leads to oxidative stress and free radical production which can damage DNA, proteins, and lipids. Ferroptosis, a form of regulated cell death that is dependent on iron and ROS, is characterized by the accumulation of ROS from iron metabolism, NAPDH oxidase activity and lipid peroxidation products [20]. Ferroptosis sensitivity has been shown to be modulated by NCOA4. NCOA4 deletion inhibits ferroptosis by blocking ferritinophagy and ferritin degradation, while NCOA4 over-expression increases sensitivity to ferroptosis [19]. Dysregulation of iron metabolism and ferroptosis can play a role in infection contributing to the pathogenesis of the disease.

## 3. Iron in Infection

### 3.1. Role of Iron in Microorganism Pathogenicity

Iron is an essential element for both microbes and humans. The host employs mechanisms to sequester iron, while the pathogens attempt to circumvent these withholding strategies. Bacterial invaders that can scavenger iron from the host have the potential to cause infection. Prokaryotes have two highly conserved iron-responsive regulators that respond to iron deprivation. These regulators are able to repress transcription of iron acquisition genes when the intracellular iron concentration is high and dull this repression when concentrations are low. For Gram-negative bacteria, iron homeostasis is controlled by members of the ferric uptake regulator (Fur) superfamily. Gram-positive bacteria utilize the diphtheria toxin repressor (DtxR) to control virulence gene expression and iron uptake [21]. In several bacterial genera including *Escherichia, Salmonella, Klebsiella, Yersinia,* and *Vibrio*, Fur controls iron acquisition via the small regulatory non-coding RNA (sRNA) RyhB. Under iron-rich conditions, Fur acts as a negative regulator of RyhB and iron uptake genes by binding within the promoter region of target genes, preventing their expression. Contrarily, when iron availability is low, Fur becomes inactive and the production of and RyhB iron acquisition systems is initiated [22]. The role of Fur and RyhB in the virulence of human pathogens has been widely studied. Studies by Porcheron et al. (2014) found that in a murine model of urinary tract infection, a RyhB mutant and a double RyhB*fur* mutant significantly reduced bladder colonization of *Escherichia coli* [23]. Furthermore, studies by Reinhart et al. (2015) used a murine model of acute lung infection to demonstrate that mice infected with an iron-responsive PrrF1 and PrrF2 sRNA mutant survived during the entire 28-day course of the experiment, while mice infected with the wild-type strain succumbed to lung infection [24].

Fur and RyhB control an arsenal of virulence factors that allow bacteria to invade eukaryotic cells and resist environmental stresses. Iron and heme acquisition systems are directly repressed by Fur in iron-replete environments for both Gram-negative and Gram-positive bacteria [22]. Fur also plays a role indirectly in the regulation of iron acquisition. Fur represses expression of pvdS, which directly activates expression of genes for pyoverdin siderophore biosynthesis and uptake [25]. Furthermore, RyhB regulation of iron acquisition and homeostasis is well established in *E. coli* strains. In the uropathogenic *E. coli* strain CFT073 a RyhBmutant resulted in a reduced biosynthesis of all three types of siderophores produced by this strain [23].

Additionally, invading pathogens have evolved mechanism to access heme from intracellular hemoproteins through the secretion of hemolysins. Expression of these cytolytic factors is often induced under conditions of iron starvation. A number of bacterial pathogens are able to acquire extracellular heme as a cofactor or iron source through the synthesis and secretion of small, soluble, heme-binding proteins known as hemophores. Hemophores are able to capture both free heme and appropriating it from hemoproteins. Two major types of hemophores have been characterized: HasA-type hemophores of Gram-negative pathogens and the near iron transporter (NEAT)-domain containing hemophores of Gram-positive bacteria [21]. However, some bacteria such as *S. aureus* are not known to use hemophores; thus, heme extraction, binding and transfer are all performed by cell surface-associated proteins. *Staphylococcus aureus* utilizes the Isd pathway consisting of nine iron-regulated proteins, IsdA through IsdI. This pathway is required for uptake of iron from physiological concentrations of heme and the heme-degrading enzymes IsdG and IsdI are required for maximal virulence in murine models of infection [26,27].

### 3.2. Siderophores

**Figure 2:**
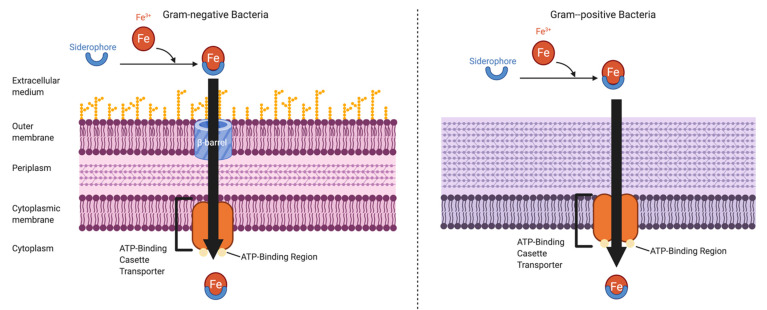
An overview of iron-loaded siderophore transport in Gram-negative compared to Gram-positive bacteria. Gram-negative bacterial cell wall is composed of an outer and inner membrane, separated by the periplasm. Gram-negative bacteria recognize iron-loaded siderophores through a through a β-barrel receptor in the outer membrane. Ligand binding results in a conformational change translocating the iron-loaded siderophore into the periplasm. Transport into the cytoplasm and iron reduction is mediated by ABC transporter in the inner membrane. In Gram-positive bacteria, the iron-loaded siderophore can be directly imported into the cytosol via ABC transporters. Figure generated with bioRender.

Bacteria acquire iron by secreting siderophores, small ferric iron-binding molecules. Affinities of bacterial siderophores to iron are generally much higher than those of host proteins. This allows bacteria to outcompete the host in the battle over limited iron [28]. Excreted siderophores bind available ferric iron forming a ferri-siderophore complex. This complex will then bind to specific receptor proteins present on the microbial cell surface and become internalized via active transport. The mechanisms of uptake of iron-loaded siderophores differs between Gram-negative and Gram-positive bacteria. Gram-negative bacteria recognize iron-loaded siderophores through a β-barrel receptor in the outer membrane. Ligand binding results in a conformational change, translocating the iron-loaded siderophore into the periplasm. Transport into the cytoplasm and iron reduction is then mediated by an ATP-binding cassette (ABC) transporter in the inner membrane. In Gram-positive bacteria siderophores can be directly imported into the cytosol via ABC transporters as there is no outer membrane (Figure 2). Siderophore synthesis, release and uptake mechanism are tightly regulated in order to maintain iron homeostasis within the cell [29].

Siderophores have been classified into three main groups based on their chemical structure and properties: hydroxamate, catecholate, and carboxylate. Hydroxamate siderophores are hydrophilic and are the most common group of siderophores in nature. Catecholate siderophores are found only in bacteria and consist of catecholate and hydroxyl groups. Compared to carboxylate and hydroxamate siderophores, catecholate siderophores have the highest affinity for ferric iron under physiological conditions. Enterobactin, primarily produced by *E. coli,* is a catechol siderophore with the highest affinity towards ferric iron than any other known siderophore [30].

### 3.3. Synthetic Iron Chelators

Siderophores can function as competitive agents against the host which aid bacteria in causing infections. In iron-limited environments siderophores can lock iron away from the host. Humans do not have their own siderophores to compete with bacteria. However, considerable progress has been made in investigating iron chelators, which are now available clinically. Desferrioxamine (DFO) is a clinically approved iron chelator originating from *Streptomyces pilous.* DFO is a chain-like molecule which can wrap around iron in a 1:1 Fe^3+^ /DFO complex (Table 1). However, DFO is not ideal for use in bacterial infections as many bacterial species are able to utilize the iron sequestered within DFO. DFO also requires prolonged i.v. infusions five to seven days a week. Research has therefore moved towards creating synthetic iron chelators which can be self-administered orally [31].

Deferiprone (DFP) is an FDA-approved oral iron chelator with comparable efficacy to DFO (Table 1). DFP is rapidly absorbed, with a peak blood level at 45 min after ingestion. Three daily doses of DFO is the current widely adopted recommendation [32]. Richter et al. (2017) studied the antibiofilm activity of a surgical wound gel loaded with DFP on multidrug-resistant bacteria. When DFP was used in combination with ciprofloxacin the efficacy exceeded the activity of the individual compound and showed significant antibiofilm activity against *Staphylococcus aureus* and *Pseudomonas aeruginosa* [33].

Deferasirox (DFX) is an FDA-approved oral iron chelator with similar efficacy to DFO (Table 1). DFX reaches peak plasma concentration within one and a half to four hours after oral administration. DFX has a half-life ranging from 8 to 16 h, allowing for once-daily dosing [34]. Studies by Puri et al. (2019) found DFX to reduce *Candida albicans* invasion of oral epithelial cells and infection levels in a murine model of oropharyngeal candidiasis. *C. albicans* cells were found to have a twofold reduction in survival and reduced adhesion to and invasion of oral epithelial cells in vitro when treated with DFX [35].

Lastly the novel iron chelator, DIBI, has shown antibacterial effects in recent studies. Thorburn et al. (2017) studied the impact of DIBI on bacterial proliferation in a murine model of abdominal sepsis. They found a significant decrease in bacterial counts in the blood and peritoneal lavage fluid (PLF) when DIBI was used in combination with imipenem [36]. Furthermore, studies by Parquet et al. (2019) demonstrated the antibacterial effects of DIBI in vitro and in experimental pneumonia in mice. An intranasal dose of DIBI after intranasal challenge with hypervirulent ciprofloxacin (CIP)-resistant *A. baumannii* significantly reduced bacterial burden in mice. DIBI was shown to restrict host iron availability and work as an anti-infective or in combination with antibiotics for the treatment of *A. baumannii* pneumonia [37].

## 4. Local Infection

### 4.1. Infections of the Eye

Infection of the eye are considered to be particularly problematic, since poorly managed infections can have devastating consequences including blindness. The eye harbors its own defense mechanisms against infection, e.g., through the production of lactoferrin (Lf). Lf is an 82 kDa protein that is produced by the acinar cells of the lacrimal glands. Lf binds free iron in the extracellular space [13]. As a result, iron is not available for pathogen use. The existence of this natural mechanism for iron restriction within the eye suggests a potential role for iron chelation in the treatment of eye infections.

Uveitis is the inflammation of the middle layer of the eye. Uveitis has a prevalence of 115.3 per 100,000 and may be either infectious or non-infectious [39]. A study by N. Arora et al. (2018) showed a promising role for iron chelator DIBI in experimental endotoxin-induced uveitis [40]. Rao et al. (1986) showed similar findings when using the iron chelator deferoxamine (DFO). DFO treatment of experimental uveitis in rats significantly reduced choroidal inflammation and suppressed retinal damage [41]. Lastly, studies by Lennikov et al. (2014) examined echinochrome as a potential therapy for endotoxin-induced uveitis (EIU) in rats. Echinochrome is a pigment found in the shells and spines of sea urchins. It has been proposed to have protective mechanisms as it is a naturally occurring iron chelator and free radical scavenger [42]. Lennikov and colleagues found echinochrome significantly reduced inflammatory cell infiltration and protein levels in the aqueous humor and ROS production in ocular tissue of EIU rats [43].

Microbial keratitis (MK) is a condition that is caused by a variety of microbes that infect the cornea and produce an inflammatory response. The predisposing factors for MK are contact lens wear, corneal injury, or ocular surface disease. It has been estimated that the incidence of MK ranges from 1 to 20 per 10,000 users depending on the type of contact lens worn [44]. MK caused by contact lens wear is most commonly associated with gram-negative bacteria *Pseudomonas aeruginosa,* a species known for biofilm formation [12]. Bacterial biofilm formation on contact lenses and their storage cases has been implicated in the pathogenesis of MK since biofilms prolong the retention time of organisms at the ocular surface and provide intrinsic antibiotic resistance [45].

**Figure 3:**
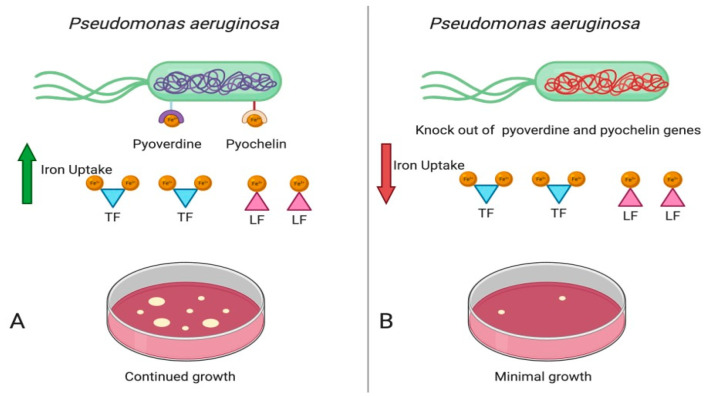
(**A**) *P. aeruginosa* produces two types of siderophores in iron limited conditions, pyoverdine and pyochelin. These siderophores have high affinity for iron and acquires iron from host transferrin and lactoferrin. (**B**) By knocking out the genes for pyoverdine and pyochelin, *P. aeruginosa* growth is inhibited. Figure generated with bioRender.

As reviewed by Kang and Kirienko (2018) iron acquisition is closely linked with both biofilm formation and pathogenicity of *P. aeruginosa* [46]. *P. aeruginosa* produces two types of siderophores in iron-limited conditions; pyoverdine and pyochelin. These siderophores acquire iron from host transferrin and lactoferrin and are necessary for the development of biofilms [46], Studies by Suzuki et al. (2018) demonstrated that *P. aeruginosa* strains defective in pyoverdine genes had significantly decreased invasion capacity and bacterial growth [47] (Figure 3). Banin et al. (2005, 2006) showed the loss of pyoverdine, but not pyochelin, was able to disrupt biofilm formation [48,49]. Additional studies have noted the anti-biofilm effects of iron chelation.

### 4.2. Skin Wound Infections

In North America currently the most common microbial strain found in wound infections is *Staphylococcus aureus* (84%), of which about 50% are methicillin-resistant *S. aureus* (MRSA) [50,51]. *S. aureus* colonizes the skin and mucosal surfaces such as the nares in 30% of healthy humans hence it is easily exposed to skin wounds [52]. Antibiotics are used to treat patients infected with *S. aureus*; however, antibiotic resistance is increasing.

**Figure 4:**
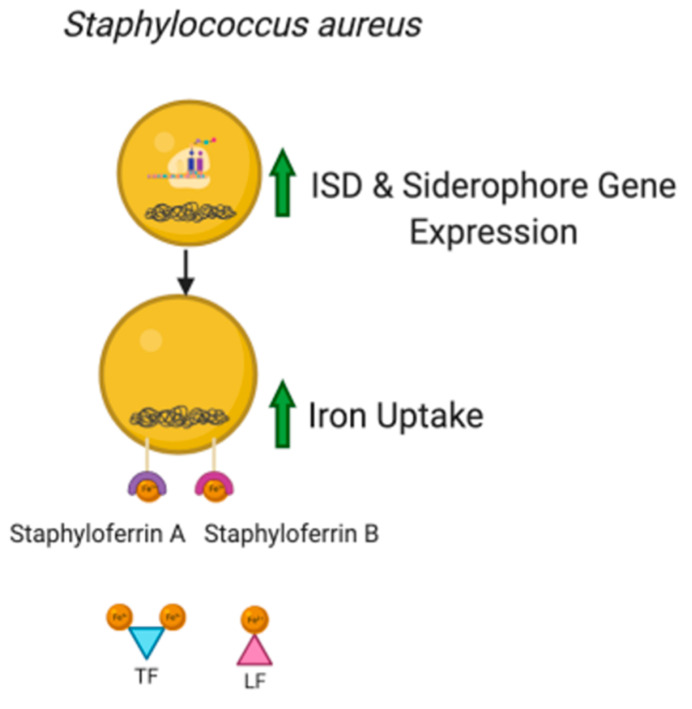
During an infection, the host’s defense system withholds iron. *S. aureus* has an iron-regulated surface determinant (Isd) locus which consists of seven genes (IsdA, IsdB, IsdC, IsdE, IsdF, IsdG, IsdH) that encode for proteins that acquire iron from heme and hemoglobin into the cytoplasm. During an infection Isd gene expression increases as well as gene expression of iron-scavenging siderophores, staphyloferrin A and staphyloferrin B. These siderophores aid *S. aureus* acquire iron from transferrin (TF) and lactoferrin (LF) as they have a higher affinity for iron than host iron binding proteins. Figure generated with bioRender.

*S. aureus* requires iron for pathogenicity. It has an iron-regulated surface determinant (Isd) locus which consists of seven genes (IsdA, IsdB, IsdC, IsdE, IsdF, IsdG, and IsdH) that encode for proteins that have been found to acquire iron from heme and hemoglobin into the cytoplasm [26]. The ferric uptake repressor (Fur) which is a DNA sequence that is found in the transcriptional units of Isd inhibits transcription when iron concentrations are high [53,54]. Fur has also been found to play a role in the biosynthesis of siderophores, staphyloferrin A and staphyloferrin B. These siderophores aid *S. aureus* acquire iron from transferrin and lactoferrin as they have a higher affinity for iron than host iron binding proteins [55]. During an infection, the host’s defense system withholds iron, and it has been suggested that Isd gene expression is high at this time (Figure 4). Moreover, in an experiment, expression of Isd was found to be higher in an *S. aureus* culture with iron chelators compared to one with *S. aureus* in a media containing iron [26]. Therefore, there is a need for synthetic iron chelators that can aid in iron sequestration.

The novel iron chelator DIBI, a hydroxypyridinone-containing iron-chelating antimicrobial polymer, has been shown as an effective treatment for wound infections caused by *S. aureus*. Studies by Parquet et al. (2018) found DIBI to be strongly inhibitory to a diverse group of *S. aureus* isolates (human, cattle, and dog). Additionally, antibiotic-resistant hospital and community-acquired MRSA isolates were also inhibited by DIBI. Furthermore, topical application of DIBI to a skin wound infection provided a dose-dependent suppression of infection and reduced inflammation. Lastly, DIBI/antibiotic interactions were studied and DIBI did not impair the killing activity of 274 antibiotics, and actually increased the initial or extended the initial antibiotic killing phase [52]. Results of these studies display the potential of iron chelators to work in conjunction with antibiotics to improve their effectiveness.

### 4.3. Bacterial Cystitis

Bacterial cystitis (BC), commonly known as urinary tract infection (UTI), is among the most common bacterial infections affecting 150 million people worldwide each year [56]. BC occurs most commonly in otherwise healthy women when uropathogenic bacteria, most commonly *Escherichia coli* (UPEC) from the gastrointestinal flora, enters the urethra and ascends into the bladder [57]. Approximately 25% of women who experience bacterial cystitis will suffer a recurrent UTI within six months of the initial episode. Trimethoprim-sulfamethoxazole (TMP-SMX), nitrofurantoin, and Fosfomycin are the current treatments of choice for cystitis in women. However, antimicrobial resistance among uropathogens has been increasing to TMP-SMX and antibiotics fail to eliminate recurrences [58].

Autophagy commonly acts as a host defense mechanism against invading pathogens. During this process, damaged organelles, proteins, and invading microbes are broken down and recycled via fusion of autophagosomes and lysosomes. Ferritinophagy is a form of selective autophagy important in iron homeostasis. Host cells utilize this pathway to recycle iron by shuttling iron-bound ferritin to lysosomes [59]. *Escherichia coli* persists within the urinary tract epithelium (urothelium) by forming reservoirs within autophagosomes [60]. Studies by Bauckman and Mysorekar (2016) reported that UPEC utilizes host ferritinophagy in order to shuttle with ferritin-bound iron into the autophagosomal and lysosomal compartment of the urothelium [59]. Their study demonstrated that excess iron increased intracellular UPEC growth in a dose-dependent manner. In addition, iron chelation treatment with DFO decreased bacterial growth in urothelial cells and improved host cell survival [59]. These results were further investigated by Bauckman et al. (2019) using a UPEC induced mouse model of BC. Treatment with a low iron diet decreased local bladder tissue iron stores and reduced bacterial colonization and inflammation. Furthermore, hepcidin-deficient mice (*Hamp1^−/−^)* exhibited an accumulation in iron deposits and significantly higher bacterial colonization and heightened inflammatory response to the UTI [61]. Lastly, studies by Hagan and colleagues (2010) also demonstrate the importance of iron in UPEC pathogenesis. In urine samples from women with cystitis, genes involved in siderophore production and iron acquisition were the most highly expressed virulence determinants across all isolates. Furthermore, pathogen-specific genes were found to be expressed during human cystitis. UPEC isolates produced siderophores that are not synthesized by most non-pathogenic fecal *E. coli* strains suggesting horizontally acquired fitness genes [62]. Results of these studies demonstrate the potential for iron chelators as treatment for bacterial cystitis.

### 4.4. Medical Device-Associated Infections

Medical device-associated infections result from the use of a foreign object within the body for medical purposes. These infections are commonly associated with indwelling devices such as urinary catheters and prosthetic joints, and account for approximately half of all healthcare-associated infections [63]. The presence of these objects facilitates the formation of biofilms, which consist of bacteria which adhere to both each other and a surface. These bacteria are contained within a matrix consisting of polysaccharides and other materials. Infections related to biofilm formation are typically difficult to resolve due to the adaptations facilitated by biofilm formation. Biofilms exhibit altered gene expression, protein production, and metabolism, and have an increased level of both antibiotic resistance and resistance to the host immune response [64]. Additionally, patient populations with indwelling devices tend to be aging and faced with various comorbidities, which compromises the immune system. Device-associated infections have variable mortality, depending on the type of device and infecting pathogen. For example, catheter associated UTIs have a mortality rate of approximately 5%, while infections of prosthetic hip joints have a mortality rate of 7% [65,66].

Urinary catheters are common indwelling devices and are often used long-term. Patients develop a chronic, asymptomatic bacteriuria after just several weeks of catheter usage [67]. In a European prevalence study, only 1.3% of patients went on to develop a symptomatic infection [68]. Gram-negative Enterobacteriaceae, and non-Enterobacteriaceae, such as *P. aeruginosa,* are common uropathogens in patients with indwelling catheters [69]. These pathogens, specifically *E. coli* and *P. aeruginosa*, are known biofilm formers, which complicates treatment. Most infections are considered avoidable with proper sterile technique and appropriate management, but there is still a need for new strategies to manage these complex infections. Because of the critical role of iron in biofilm formation and bacterial pathogenesis, iron chelation may be able to play a role in infection management. Catheters made of iron-scavenging materials may reduce biofilm formation, but this concept has yet to be tested clinically. Adding physiological chelator lactoferrin to *P. aeruginosa* cultures reduced biofilm formation [70]. In combination with colistin, iron chelation with a synthetic hexadentate chelator is able to almost completely eradicate *P. aeruginosa* biofilms [71]. An alternative strategy involves the addition of chelating agents to catheter lock solutions. Chelating agents such as EDTA and citrate have shown anti-biofilm activity in the context of lock solutions for central venous catheters but are not currently approved for use in Canada [72].

Joint replacement is a frequent procedure, with the number of annual procedures rising along with an aging population. In the USA, the number of annual hip or knee arthroplasties is expected to reach 4 million by 2030 [73]. The rate of prosthetic joint infection following surgery is 2% and increases with revision surgery [74]. The most commonly isolated pathogens include *S. aureus*, coagulase-negative Staphylococcus (CNS), Enterococcus, and Gram-negative bacilli [74]. As reviewed by Hall-Stoodley et al. (2012), it is challenging to directly identify biofilm-related infections as standard culture methods are insufficiently sensitive to detect pathogens within a biofilm [75]. Systemic antibiotics successfully eradicate planktonic bacteria while leaving the biofilm intact. As such, biofilm-related infections are often culture-negative despite clinical signs of infections. Iron chelation may enhance the penetration of antibiotics into biofilms. Combination therapy, with both deferiprone (DFP) and conventional antibiotics, showed enhanced activity against CNS biofilms on titanium [76]. Recent advances in this field include the development of a novel polycyclic polyprenylated acylphloroglucinol antibiotic with mild chelating activity, giving it enhanced anti-microbial and anti-biofilm activity [77].

### 4.5. Iron in Viral Infection

In order to efficiently replicate and infect host cells, viruses require iron for fundamental cellular processes such as DNA synthesis and ATP production. Individual viruses have different mechanisms of invasion. A primary initiating event involves the interaction of the virus with a receptor or set of plasma membrane surface markers. TfR1 is highly expressed, making it a target for the virus to recognize and bind to [78]. Canine parvovirus (CPV) and feline panleukopenia virus (FPV) were the first pathogens recognized to infect cells through TfR1 trafficking pathways. Studies by Parker et al. (2001) found that anti-TfR1 antibodies were found to block CPV infection [79] Additionally, Radoshitzky et al. (2007) demonstrated a specific, high affinity association between TfR1 and the entry glycoprotein (GP) of Machupo virus, a New World hemorrhagic fever arenavirus. They found expression of human TfR1 in hamster cell lines enhanced the infection of viruses with the GP of Machupo and an anti-TfR1 anti-body efficiently inhibited the replication of the virus. Furthermore, iron depletion of culture medium enhanced the efficiency of infection while iron supplementation decreased this efficiency of infection, demonstrating that TfR1 is a cellular receptor for New World hemorrhagic fever arenaviruses [80].

Host iron status has been described to affect the induction and propagation of viral mutations, mainly through mechanisms of oxidative stress. Redox regulation can lead to faster viral replication and direct oxidative damage to genomic alterations [78]. A number of viral infections, including viruses of the *Flavivirus* genus, have been found to trigger oxidative stress. Therefore, the maintenance and restoration of a homeostatic intracellular environment is crucial for the host to combat viral infection. Among Flaviviridae viruses, hepatitis C virus (HCV) infection and the induction of oxidative stress has been more extensively studied. Approximately 170 million people worldwide are infected with HCV, which leads to chronic liver disease. It is widely accepted that chronic hepatitis C (CHC) is associated with iron overload and hepatic iron accumulation. HCV alters iron metabolism by reducing hepcidin levels [81]. Almost all HCV proteins have been demonstrated to be involved in the induction of oxidative stress. Antioxidant defenses work to attenuate cellular oxidative stress and return the cell to a basal state. However, these antioxidant systems are manipulated by Flaviviridae viruses and are associated with chronic HCV infection. Mitochondria are the major source of ROS inside hepatocytes and liver-resident blood cells. Excessive ROS production is the leading factor that contributes to liver inflammation, fibrogenesis, and hepatic carcinogenesis [82]. Studies by Yano et al. (2007) found β-carotene, vitamin D, and linoleic acid inhibited HCV RNA replication and that their combination caused additive effects. However, the use of antioxidants as therapeutics against HCV remains controversial as results from Yano and colleagues also found vitamin E to enhance HCV RNA replication [83].

## 5. Therapeutic Applications

### 5.1. Routs of Administration

In order to be used therapeutically, synthetic iron chelators must be able to compete with biological iron binding substances. Most of the modern iron chelators can be administered topically for local infection and inflammation, e.g., in eye drops or creams [40]. However, the oral bioavailability of some novel iron chelators, such as DIBI, is limited. In the case of colitis, the iron chelator maltol can be administered orally [84]. Furthermore, although DFO has been shown as a potential treatment for both local infections and inflammation it requires prolonged infusion five to seven days per week. FDA-approved iron chelators, DFP and DFX can both be administered as an oral tablet and have longer half-lives, 3–4 h and 8–16 h, respectively [38,85]. DFP and DFX have comparable efficacy to DFO and can be self-administered one to three times daily. Future research should therefore examine the potential role of these iron chelators in treating local infections and inflammation.

### 5.2. Limitations

Depleting iron from the host is beneficial in reducing local infection. However, the local administration of iron chelators should be limited in order to avoid systemic effects. Moreover, theoretically, local iron restriction could have an impact on host immune response by attenuation of local ROS production. During pathologic states, unshielded labile iron accumulates in plasma and is readily taken up by hepatocytes and other tissue parenchymal cells. Exposure of proliferating cells to different concentrations of H_2_O_2_ triggers a range of responses including increased proliferation rates, permanent inhibition of cell proliferation and apoptotic or necrotic cell death. There is evidence that chelation of intracellular labile iron inhibits H_2_O_2_-mediated expression of adhesion molecules and the resulting recruitment of monocytes [86]. Labile iron plays a crucial role in the signaling mechanisms that differentiation between survival or death in cells exposed to H_2_O_2_. Furthermore, H_2_O_2_ can stimulate iron sequestration within ferritin, mitigating post-transcriptional ferritin suppression [87]. The presence of multiple opposing mechanisms for ferritin regulation underlies the fine line necessary for regulating the labile iron pool in response to a variety of stimuli.

Therefore, pharmacological modulation of labile iron by chelation therapy is critical for the management balance between prevention of cell damage and meeting the cellular demands. In general, the local administration of iron chelators represents a safe pharmacological approach with a low side effect profile.

## 6. Conclusion

Iron plays a crucial role in the host’s innate immune response and iron balance is necessary in order to treat infections. Bacteria with enhanced iron uptake mechanisms (e.g., *Pseudomonas aeruginosa* and *Escherichia coli)* have been shown to have increased virulence. Excess iron has been shown to exacerbate infection in microbial keratitis, skin wound infections and bacterial cystitis. Therefore, depriving bacteria of iron through synthetic iron chelators which acts to enhance the host’s innate iron-withholding mechanisms presents as a potential treatment for local infections. As shown through the examples above, during local infection depletion of iron can be effective in reducing microbial proliferation. This is the rationale for the use of iron chelators under those conditions.

## Figures and Tables

**Table 1:** Overview of selected iron chelators [38].

Properties	Desferrioxamine (DFO)	Deferasirox (DFX)	Deferiprone (DFP)
Binding capacity (chelator: iron)	Hexadentate (1:1)	Bidentate (2:1)	Tridentate (3:1)
Route of administration	Subcutaneous, intravenous	Oral tablet	Oral tablet
Side effects	Growth retardation Local skin reaction Ophthalmological Auditory Allergic reaction Pulmonary at high doses Neurological at high doses	Rash Rise in creatinine Auditory Gastrointestinal Ophthalmological	Gastrointestinal Zinc deficiency Agranulocytosis Musculoskeletal and joint pains
Half-life	47–134 min	8–16 h	3–4 h
Structure